# A multicenter study of long-term outcomes of relapsing polychondritis in Iran

**DOI:** 10.1038/s41598-024-67530-8

**Published:** 2024-07-17

**Authors:** Mehdi Jafarpour, Maryam Saberivand, Maryam Saemi, Maryam Sahebari, Seyedmostafa Seyedmardani, Mansour Salesi, Sarah Hosseinpoor, Tahereh Faezi, Kamal Esalatmanesh, Mehrzad Hajialilo, Sousan Kolahi, Zahra Myrfeizi, Alireza Khabbazi

**Affiliations:** 1 Connective Tissue Diseases Research Center, Tabriz University of Medical Sciences, Golgasht St., Tabriz, Iran; 2 Rheumatic Diseases Research Center, Mashhad University of Medical Sciences, Mashhad, Iran; 3 Department of Internal Medicine, Urmia University of Medical Sciences, Urmia, Iran; 4 Department of Internal Medicine, Isfahan University of Medical Sciences, Isfahan, Iran; 5 Rheumatology Research Center, Tehran University of Medical Sciences, Tehran, Iran; 6 Department of Internal Medicine, Kashan University of Medical Sciences, Kashan, Iran; 7 Department of Internal Medicine, Mashad University of Medical Sciences, Mashhad, Iran

**Keywords:** Relapsing polychondritis, Remission, Disease modifying antirheumatic drugs (DMARDs), Relapse, Immunosuppressants, Immunology, Rheumatology

## Abstract

Relapsing polychondritis (RP) is a systemic immune mediated disease characterized by recurrent episodes of inflammation in various cartilage-rich areas. RP may cause extensive tissue destruction and is associated with significant morbidity and mortality. In this multicenter study, we considered the remission status and long-term outcomes of RP in patients who were followed-up in six referral rheumatology centers in Iran. Outcomes of disease was assessed by remission status and RP induced damage. A total of 29 patients with RP were examined for enrollment in the study, and 26 patients with a minimum follow-up period of 6 months were included in the RP outcome analysis. Median time to control of symptoms and sustained remission were 5 and 23 weeks, respectively. Prednisolone was discontinued in 8 (30.8%) patients and medication-free remission was achieved in 7 (23.1%) patients. Regarding the disease course, 34.6% of patients had a relapsing–remitting course, 42.3% had a monophasic course, and 23.1% had an always-active course. Despite extensive treatment with immunosuppressive medications, RP induced damage was developed in 21 (80.8%) patients. Ear deformity and osteoporosis were the most common RP induced damage. Long-term remission and medications-free remission in RP is accessible. However, RP related damage occur in majority of patients.

## Introduction

Relapsing polychondritis (RP) is a systemic immune mediated disease characterized by recurrent episodes of inflammation in various cartilage-rich areas, such as the ears, nose, joints and respiratory tract^1^. Although the exact cause of RP is unknown, it is thought to be an autoimmune disorder, as circulating autoantibodies against collagens II, IX, XI, cartilage oligomeric matrix proteins, and matrilin-1 have been identified in these patients^2^. RP is a rare disease, with an estimated prevalence of 3.5–4.5 cases per million people^2^. It can affect peoples of any age, but it most commonly presents in ages of 40 and 60, and both males and females can be affected equally^2,3^.

RP may cause extensive tissue destruction and is associated with significant morbidity and mortality^1,2^. Considering the relapsing remitting course of RP, the goal of treatment in this disease is to control the inflammatory crisis to prevent further tissue damage and long-term suppression of immune-mediated pathogenic mechanisms to prevent disease recurrence^2,4^. Due to the rarity of the disease and lack of controlled studies, there are no evidence-based guidelines for the treatment of RP. However, non-steroidal ant-inflammatory drugs (NSAIDs) and glucocorticoids (GCs) used for control of acute inflammation and conventional synthetic disease modifying antirheumatic drugs (csDMARDs) including azathioprine, methotrexate, cyclophosphamide, and cyclosporine and biologic DMARDs (bDMARDs) used for control of immune system activity with variable results^2,4^.

Despite numerous case reports and case series on the clinical manifestations and treatments of RP, there are few data on the long-term outcomes of RP treatment. In this multicenter study, we considered the remission status and long-term outcomes of RP.

## Methods

### Study population

This retrospective multicenter study was conducted to investigate the long-term outcomes of RP in patients who were followed-up from July 2014 to October 2023 in six referral rheumatology centers in Iran including, Tabriz, Mashhad, Urmia, Tehran, Kashan, and Isfahan universities of medical sciences. Patients were included in the study if (i) were older than 18 years at disease onset, (ii) met the McAdam's criteria^5^ modified by Damian and Levine^6^ for RP, (iii) had at least 3 visits per year and (iv) had at least 6 months follow-up. Patients with insufficient data, irregular follow-up and loss to follow-up were excluded. The study protocol was approved by the ethics committee of Tabriz University of Medical Sciences (Ethical code: IR.TBZMED.REC.1402.294). Informed consent was obtained from all participants. This study was conducted in accordance with the Declaration of Helsinki.

### Data collection and outcome assessment

The demographic, clinical, laboratory, therapies and outcomes data of the patients were obtained from their charts. The follow-up time was determined from the date of entering the cohort to the last visit. In case of incomplete data or loss to follow-up, we tried to obtain information through direct or telephone interviews. Disease activity was assessed using the Relapsing Polychondritis Disease Activity Index (RPDAI)^7^. Remission in all patients was assessed by an expert rheumatologist. Outcomes of disease was assessed by remission status and RP induced damage. Control of symptoms was defined as control of inflammatory symptoms (chondritis, arthritis, skin lesions, fever, etc.) with any dose of prednisolone and DMARDs for at least 4 weeks. Sustained remission was defined to control of inflammatory symptoms, prednisolone dose ≤ 7.5 mg/d for at least 12 weeks. DMARDs were permitted. The control of the inflammatory symptoms in chondritis was defined as the disappearance of pain, swelling and erythema, which was checked by examination and, if necessary, bronchoscopy and imaging. Arthritis control was defined as the absence of pain and swelling in the joint as assessed by a clinician. Relapse of RP was defined as (1) worsening or development of new RP-related symptoms identified by physical examination or imaging studies and leading to treatment escalation or (2) an increase in C-reactive protein (CRP) and erythrocyte sedimentation rate from baseline levels, which is considered due to RP activity and led to treatment intensification^8^. In patients with multiple remissions and relapses, the remission with the longest duration was used in the analysis of duration of remission. RP induced damage was assessed using the Relapsing Polychondritis Damage Index (RPDAM)^9^.

### Statistical analysis

Statistical analysis was performed using SPSS software version 16.0 (SPSS, Inc., USA). The normal distribution of data was assessed using the Kolmogorov–Smirnov test. Normally and non-normally distributed continuous variables were reported as mean ± standard deviation (SD) and median (25–75% interquartile range [IQR]), respectively. Categorical variables were reported as frequency and percentage. Kaplan–Meier test was used for survival analysis. *P*-values less than 0.05 were considered as statistically significant.

## Results

A total of 29 patients with RP were examined for enrollment in the study, whose demographic, clinical and laboratory characteristics are shown in Table 1. Finally, 26 patients with a minimum follow-up period of 6 months were included in the RP outcome analysis (Table 2). Mean age of the participants at the time of diagnosis was 42.8 ± 15.3 years and female:male ratio was 1.6. The median (IQR) duration of follow-up was 41 (19, 73) months. Auricular chondritis, nose chondritis, laryngeal chondritis, fever, arthralgia/arthritis, scleritis and uveitis were the most frequent clinical manifestations (Table 1). At least one auto-antibodies including perinuclear antineutrophilic cytoplasmic antibody (P-ANCA), cytoplasmic ANCA (C-ANCA), anti-nuclear antibody (ANA) and rheumatoid factor (RF) was positive in 17 (58.6%) patients (Table 1).

**Table 1:** Demographic, clinical and paraclinical characteristics of included patients (n = 29).

Demographic characteristics	
Age at the time of diagnosis, mean ± SD, years	42.8 ± 15.3
Female (%)	18 (62.1)
Familial history of rheumatic disease (%)	4 (13.8)
Smoking (%)	3 (10.3)
Disease duration before diagnosis, median (IQR), weeks	22 (10.5, 64.0)
Chondritis (%)	29 (100)
Auricular Chondritis (%)	25 (86.2)
Nasal chondritis (%)	16 (55.2)
Respiratory tract chondritis (%)	12 (41.4)
Ocular inflammation (%)	11 (37.9)
Scleritis (%)	4 (36.4)
Uveitis (%)	4 (36.4)
Keratitis (%)	2 (18.2)
Episcleritis (%)	1 (9.1)
Conjunctivitis (%)	1 (9.1)
Optic neuritis (%)	1 (9.1)
Inflammatory arthritis	7 (24.1)
Monoarthritis (%)	2 (28.6)
Oligoarthritis (%)	4 (57.1)
Polyarthritis (%)	1 (14.3)
Audio vestibular dysfunction (%)	5 (17.2)
Constitutional symptoms (%)	11 (37.9)
Renal involvement	4 (13.8)
Proteinuria (%)	3 (10.3)
Active urine (%)	3 (10.3)
Skin lesions (%)	2 (6.9)
Oral ulcer (%)	2 (6.9)
Overlap with other diseases (%)	6 (20.7)
Systemic lupus erythematosus (%)	1 (3.5)
ANCA associated vasculitis (%)	1 (3.5)
Behcet’s disease (%)	1 (3.5)
Rheumatoid arthritis (%)	1 (3.5)
Autoimmune hepatitis (%)	1 (3.5)
Laboratory parameters	
Leukocytosis (%)	4 (13.8)
ESR, median (IQR), mm/h	35 (11, 72)
High CRP (%)	23 (79.3)
ANCA-P (%)	7 (24.1)
ANCA-C (%)	1 (3.5)
ANA (%)	6 (20.7)
RF (%)	5 (17.2)

*SD* standard deviation, *IQR* interquartile range, *P-ANCA* perinuclear antineutrophilic cytoplasmic antibody, *C-ANCA* cytoplasmic ANCA, ANA anti-nuclear antibody, *RF* rheumatoid factor.

**Table 2:** Patients’ medications and outcomes of treatment (n = 26).

Duration of follow-up, median (IQR), months	41 (19, 73)
Medications	
Prednisolone (%)	25 (96.2)
Azathioprine (%)	14 (53.8)
Methotrexate (%)	13 (50.0)
Cyclophosphamide (%)	5 (19.2)
Leflunomide (%)	4 (15.4)
Mycophenolate mofetil (%)	4 (15.4)
TNF inhibitors (%)	4 (15.4)
Dapson (%)	3 (11.5)
Hydroxychloroquine (%)	2 (7.7)
Colchicine (%)	2 (7.7)
Rituximab (%)	1 (3.8)
Adherence to therapy (%)	20 (76.9)
Results of treatment	
Control of symptoms (%)	22 (84.6)
Sustained remission (%)	20 (76.9)
Resistant to therapy (%)	4 (15.4)
RPDAI at cohort entry, median (IQR)	22.5 (17.2, 30.5)
RPDAI at last visit, median (IQR)	0 (0, 13)
Time to control of symptoms, median (IQR), weeks	5 (4, 12)
Time to sustained remission, median (IQR), weeks	23 (12, 49)
Duration of remission, median (IQR), months	36.5 (12, 48)
Disease course	
Relapsing remitting (%)	9 (34.6)
Mono phasic (%)	11 (42.3)
Always active (%)	6 (23.1)
Glucocorticoids	
Initial prednisolone dose (mg/d), median (IQR)	30 (11, 30)
Final prednisolone dose (mg/d), median (IQR)	5 (0, 10)
Prednisolone discontinuation (%)	8 (30.8)
Flare of disease after prednisolone discontinuation (%)	2 (25)
Duration of prednisolone free remission, median (IQR), months	45 (12, 58)
Initial therapy	
Monotherapy with DMARDs (%)	21 (80.8)
Combination of 2 DMARDs (%)	3 (11.5)
Only prednisolone (%)	2 (7.7)
Treatment during disease course	
Continuation of initial DMARDs (%)	9 (34.6)
Changing of initial DMARDs because of inefficacy or intolerance (%)	8 (30.8)
Adding other DMARDs (%)	7 (26.9)
No DMARDs (%)	2 (7.7)
DMARDs	
Medications-free remission (%)	6 (23.1)
Relapse of disease after DMARDs discontinuation (%)	3 (50)
Time to medications-free remission, median (IQR), months	43.5 (16, 90)
Duration of medications-free remission, median (IQR), months	32.5 (7.3, 57.7)
Remission in last visit (%)	18 (69.2)
Duration of remission in last visit, median (IQR), months	38 (14, 48)
Treatment at the last visit	
Only prednisolone (%)	1 (3.8)
Prednisolone and DMARDs (%)	16 (61.5)
Only DMARDs (%)	4 (15.4)
No medications (%)	5 (19.2)

*IQR* interquartile range, *TNF* tumor necrosis factor, *RPDAI* relapsing polychondritis disease activity index, *DMARDs* disease-modifying antirheumatic drugs.

**Figure 1:**
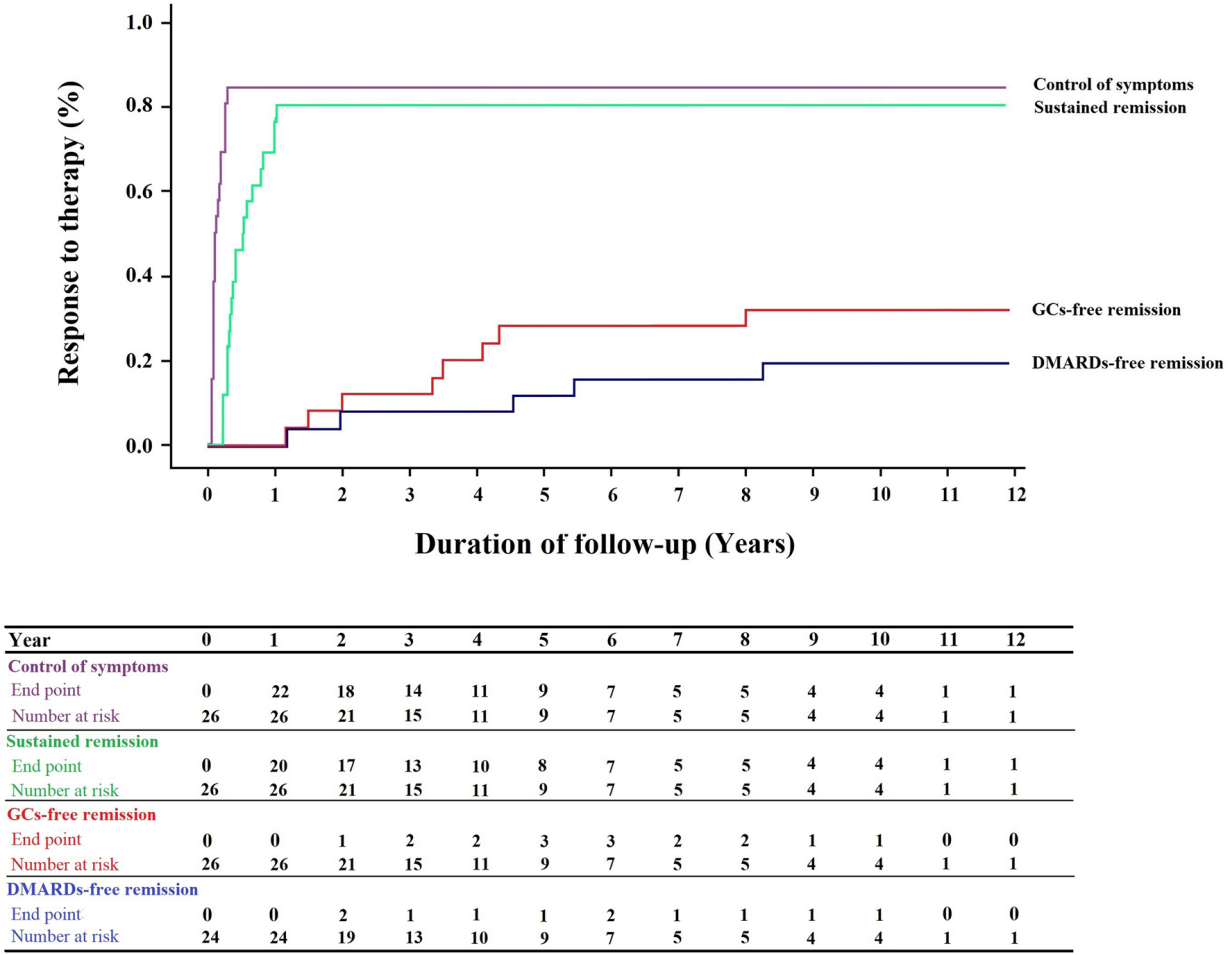
Kaplan Meier survival curve of relapsing polychondritis treatment results in the studied patients. Numbers at the bottom indicate the number of patients at risk and the number of events at each follow-up year. GCs: glucocorticoids; DMARDs: disease modifying anti-rheumatic drugs.

All patients were treated with GCs and/or DMARDs. Prednisolone was the most commonly used medication (Table 2). Other frequently used medications were azathioprine (53.8%), methotrexate (50.0%), and cyclophosphamide (19.2%). As initial therapy, all but 2 patients were treated with DMARDs. Monotherapy with DMARDs was performed in 21 (80.8%) patients and combination therapy was performed in 3 (11.5%) patients. However, during the course of the disease, a second DMARD was added to the treatment regimen in 7 (26.9%) patients, and the first DMARD was changed to another DMARD in 8 (30.8%) patients. Treatment with GCs and DMARDs lead to control of symptoms in 84.6% of patients and sustained remission in 76.9% of patients. Median time to control of symptoms and sustained remission were 5 and 23 weeks, respectively (Table 2 and Fig. 1). In all patients who responded to treatment, the response occurred within 12 months of diagnosis and initiation of treatment (Fig. 1). None of the demographic and clinical characteristics of the participants were predictive of sustained remission (Table 3). Although delay in diagnosis was shorter in patients with sustained remission, the difference did not reach significant levels (Table 3). Prednisolone was discontinued in 8 patients (30.8%) and medication-free remission was achieved in 7 (23.1%) patients. Regarding the disease course, 34.6% of patients had a relapsing–remitting course, 42.3% had a monophasic course, and 23.1% had an always-active course.

**Table 3:** Comparison of demographic and clinical characteristics of patients according remission status.

Demographic characteristics	Sustained remission (N = 20)	Active disease (N = 6)	*P*-value
Age at the time of diagnosis, mean ± SD, years	42.3 ± 14.6	40.7 ± 13.9	0.816
Female (%)	11 (55)	5 (83.3)	0.225
Smoking (%)	3 (17.6)	0	–
Disease duration before diagnosis, median (IQR), weeks	20 (10.5, 64)	52 (15, 130)	0.442
Chondritis (%)			
Ear (%)	17 (85.0)	5 (83.3)	0.676
Nose (%)	8 (40)	5 (83.3)	0.080
Larynx (%)	8 (40)	2 (33.3)	0.580
Eye involvement (%)	7 (35)	2 (33.3)	0.668
Renal involvement	2 (10)	1 (16.7)	–
Comorbidity (%)	11 (55)	3 (50)	0.596
Compliance to therapy (%)	17 (85)	3 (50)	0.252
RPDAI at cohort entry, median (IQR)	24 (13.7, 31.5)	20 (17, 30)	0.929
Initial prednisolone dose, median (IQR)	30 (15, 45)	20 (8, 30)	0.403
bDMARDs	3 (15)	2 (33.3)	–
RPDAM, median (IQR)	0.5 (0, 1)	1.5 (1, 3)	**0.016**

*SD* standard deviation, *IQR* interquartile range, *RPDAI* relapsing polychondritis disease activity index, *DMARDs* disease-modifying antirheumatic drugs, *bDMARDs* biologic DMARDS; RPDAM, relapsing polychondritis damage index.

Significant values are in bold.

Despite extensive treatment with immunosuppressive medications, RP induced damage was developed in 21 (80.8%) patients (Table 4). Sixteen (61.5%) were damaged due to the inflammatory process, 11 (42.3%) were damaged due to treatment complications and 6 (23.1%) suffered from both (Table 4). Median (IQR) RPDAM was 1 (0.8, 2). Ear deformity and osteoporosis were the most common RP induced damage (Table 4). One of the most debilitating RP induced damage is tracheobronchial which occurred in 3 patients (11.5%), and one of them required tracheostomy. We compared the demographic and clinical characteristics of patients with and without damage. Except for the higher frequency of damage in patients with trachea laryngeal involvement, none of the demographic and clinical characteristics of the participants were predictor of damage (Table 5). Surgery was performed in 3 patients (11.5%) for corneal transplant (n = 1), total hip replacement (n = 1) and tracheobronchial stenosis (n = 1). Although delay in diagnosis and time to remission was shorter in patients without damage, the difference did not reach significant levels. Damage severity assessed by RPDAM was lower in patients with sustained remission than in patients with no sustained remission (Table 3) Five years survival rate was 95.5%. One patient died during follow-up due to pneumonia.

**Table 4:** Relapsing polychondritis induced damage in various organs (n = 26).

Any damage (%)	21 (80.8)
RPDAM, median (IQR)	1 (0.8, 2)
Damage induced by inflammatory process	16 (61.5)
Ear deformity (%)	9 (34.6)
Hearing loss (%)	5 (19.2)
Saddle nose deformity (%)	4 (15.4)
Laryngotracheal stricture (%)	3 (11.5)
Corneal damage (%)	1 (3.8)
Sjogren’s syndrome (%)	1 (3.8)
Deep vein thrombosis (%)	1 (3.8)
Erosive/deforming arthritis (%)	1 (3.8)
Damage induced by treatment	11 (42.3)
Osteoporosis (%)	9 (34.6)
Avascular necrosis (%)	1 (3.8)
Severe infection needs hospitalization	2 (7.8)
Surgery for disease complications (%)	3 (11.5)
Mortality (%)	1 (3.8)

*RPDAM* relapsing polychondritis damage index, *IQR* interquartile range.

**Table 5:** Comparison of demographic and clinical characteristics of patients with and without damage.

Demographic characteristics	Any damage (N = 21)	No damage (N = 5)	*P*-value
Age at the time of diagnosis, mean ± SD, years	40.2 ± 14.8	49.1 ± 8.8	0.218
Female (%)	13 (61.9)	3 (60)	0.657
Smoking (%)	2 (9.5)	1 (20)	–
Disease duration before diagnosis, median (IQR), weeks	20 (10, 104)	46 (25, 68)	0.618
Chondritis (%)			
Ear (%)	18 (85.7)	4 (80.0)	0.600
Nose (%)	10 (47.6)	3 (60.0)	0.500
Larynx (%)	10 (47.6)	0	**0.045**
Eye involvement (%)	8 (38.1)	1 (20)	0.420
Renal involvement	2 (9.5)	1 (20)	–
Comorbidity (%)	11 (52.4)	3 (60)	0.578
RPDAI at cohort entry, median (IQR)	24 (15.5, 31)	19 (14.5, 27)	0.340
Initial prednisolone dose, median (IQR)	15 (10, 35)	30 (22, 40)	0.367
Sustained remission	15 (75)	5 (100)	0.292
Time to remission, median (IQR), weeks	48 (18, 144)	12 (10, 32)	0.115
Duration of remission, median (IQR), months	39 (21, 48)	20 (9, 51)	0.338
Follow-up duration, median (IQR), months	48.5 (27.5, 83.5)	40 (26, 108)	0.971

*SD* standard deviation, *IQR* interquartile range, *RPDAI* relapsing polychondritis disease activity index.

Significant values are in bold.

## Discussion

Management of RP poses significant challenges due to its rarity and the heterogeneity of symptoms and disease course. This study aimed to evaluate the outcomes and treatment patterns in a cohort of RP patients, shedding light on the current understanding of this complex disease. The results of this study showed that RP was diagnosed 22 weeks after the onset of symptoms and treatment with GCs and DMARDs resulted in symptom control and sustained remission in most patients (85% and 77%). Response to treatment occurred in majority of patients in 5 months and in all patients within 12 months. These results are encouraging, as achieving remission is a primary therapeutic goal in RP to prevent further damage to cartilage structures. Medication-free remission was achieved in 23% of patients. However, relapse occurred in 25% of patients after discontinuation of prednisolone and in 50% of patients after discontinuation of DMARDs, suggesting the need for ongoing monitoring and management to prevent relapse.

Although there was no significant difference in the demographic characteristics and clinical manifestations of the studied patients with previous studies^8,10–16^, it is difficult to compare treatment results with previous reports due to differences in outcome definitions and lack of data on the remission rate in most studies (Table 6). Disease activity in our study as measured by the RPDAI was 31, which is higher than the only study in which disease activity was measured by this instrument, study Yoshida et al.^8^. There was a significant difference in the treatment strategy between the different studies, so that the rate of treatment with biological drugs in the studied patients was lower than the more recent reports^8,15,16^. Despite the availability of many biological drugs in Iran, such as TNFis, rituximab and tocilizumab, the lack of approval of these drugs in the treatment of RP by insurance organizations is a possible reason for the underuse of them in studied patients. Of the 5 patients treated with bDMARDs in this report, sustained remission was achieved in 3 patients, which is comparable to the results of Sangle et al.^16^ report. However, there was no significant difference in the rate of sustained remission in patients treated with bDMARDs versus patients not treated with bDMARDs. Nevertheless, the five years survival rate in our study (95.5%) was consistent with recent studies from other countries^8,14,15^. In Shimizu et al. report GCs, csDMARDs and bDMARDs were used in 91, 60 and 14 percent of RP patients^15^. They reported increase in the prescription of csDMARDs and bDMARDs in 2019 compared to 2009 and a decrease in RP mortality from 22 to 3% during this time period^15^. Sangle et al.^16^ in a retrospective study reported a diagnosis delay of 55 weeks. Combination therapy with prednisolone and DMARDs was performed in 97% of patients and 63% eventually required biological drugs^16^. However, mortality was 18%^16^. Recent evidences shows that improvements in diagnostic methods and treatment of RP have led to earlier diagnosis and an increase in the 10-year survival rate from 55% in 1986^10^ to 91% in 2016^14^. In a single center study from Japan diagnosis delay was 22 weeks^8^. Although 26% of patients experienced sustained remission, relapse occurred in the remaining 74%^8^. Higher CRP level and monotherapy with GCs was associated with relapse^8^.

**Table 6:** Comparison of present study findings with previously published data.

	Present study	Michet et al.^10^	Kong et al.^11^	Mathew et al.^12^	Sharma et al.^13^	Dion et al.^14^	Yoshida et al.^8^	Shimizu et al.^15^	Sangle et al.^16^
Number of patients	26	112	12	43	26	142	34	190	68
Ethnic group	Caucasian	Caucasian	Asian	Caucasian (69%)	Indians	Caucasians	Asian	Asian	Caucasian (81%)
Mean age at diagnosis	43	51	34	43	45	44	49	50	44
Female (%)	62	49	75	53	63	61	50	47	68
Median disease duration before diagnosis (weeks)	22	NR	10	1664	10	52	21		55
Auricular chondritis (%)	86	85	83	88	96	89	68	83	71
Nasal chondritis (%)	55	54	33	35	81	63	15	50	79
Respiratory tract chondritis (%)	41	48	50	37	11.5	43	33	37	71
Ocular inflammation (%)	38	51	67	57	42	56	12	43	70
Audio vestibular dysfunction (%)	17	29	42	37	46	34	9	22	34
Arthritis (%)	24	52	75	60	54	68	29	47	73
Median baseline RPDAI	31	NR	NR	NR	NR	NR	25	NR	NR
Treatment with bDMARDs	19	0	0	16	NR	15	44	27	28
Remission rate (%)	77	NR	NR	NR	NR	NR	NR	NR	NR
Relapse rate (%)	45	86	NR	NR	NR	NR	74	NR	NR
Hearing loss (%)	19	12	NR	37	46	22	NR	NR	18
Saddle nose deformity (%)	15	29	17	NR	12	NR	NR	NR	14
Laryngotracheal stricture (%)	12	23	42	NR	NR	NR	NR	NR	46
CKD (%)	0	12	0	NR	NR	0	NR	NR	16
Median Duration of follow-up (months)	41	72	96	NR	NR	156	60	100	NR
Death (%)	3	NR	0	NR	8	11	6	1.6	18
Surgery (%)	11.5	NR	42	NR	11.5	8.5	17	4.2	NR

*RPDAI* relapsing polychondritis disease activity index, *NR* not reported, *bDMARDs* biologic disease-modifying antirheumatic drugs, *RPDAM* relapsing polychondritis damage index, *CKD* chronic kidney disease.

This study was a multicenter study that, to the best of our knowledge, focused for the first time on remission rates and medications-free remission in RP. However, due to the small sample size, we were unable to analyze predictors of medication-free remission.

## Conclusion

Long-term remission and medications-free remission in RP is accessible. However, RP induced damage occur in majority of patients.

## Data Availability

The data underlying this article will be shared on reasonable request to the corresponding author.

## References

[1] Lahmer T (2010). Relapsing polychondritis: An autoimmune disease with many faces. Autoimmun. Rev..

[2] Borgia F, Giuffrida R, Guarneri F, Cannavò SP (2018). Relapsing polychondritis: An updated review. Biomedicines.

[3] Horváth A (2016). A nationwide study of the epidemiology of relapsing polychondritis. Clin. Epidemiol..

[4] Arnaud L, Mathian A, Haroche J, Gorochov G, Amoura Z (2014). Pathogenesis of relapsing polychondritis: A 2013 update. Autoimmun. Rev..

[5] McAdam LP, O’Hanlan MA, Bluestone R, Pearson CM (1976). Relapsing polychondritis: Prospective study of 23 patients and a review of the literature. Medicine (Baltimore).

[6] Damiani JM, Levine HL (1979). Relapsing polychondritis-report of ten cases. The Laryngoscope.

[7] Arnaud L (2012). The relapsing polychondritis disease activity index: Development of a disease activity score for relapsing polychondritis. Autoimmun. Rev..

[8] Yoshida T (2022). Risk factors for the recurrence of relapsing polychondritis. Arthritis Res. Ther..

[9] Mertz P (2019). The relapsing polychondritis damage index (RPDAM): Development of a disease-specific damage score for relapsing polychondritis. Jt. Bone Spine.

[10] Michet CJ, McKenna CH, Luthra HS, O’Fallon WM (1986). Relapsing polychondritis. Survival and predictive role of early disease manifestations. Ann. Intern. Med..

[11] Kong KO, Vasoo S, Tay NSWT, Chng HH (2003). Relapsing polychondritis: An oriental case series. Singap. Med. J..

[12] Mathew SD, Battafarano DF, Morris MJ (2012). Relapsing polychondritis in the department of defense population and review of the literature. Semin. Arthritis Rheum..

[13] Sharma A (2014). Relapsing polychondritis: Clinical presentations, disease activity and outcomes. Orphanet. J. Rare Dis..

[14] Dion J (2016). Relapsing polychondritis can be characterized by three different clinical phenotypes: Analysis of a recent series of 142 patients. Arthritis Rheumatol..

[15] Shimizu J, Yamano Y, Kawahata K, Suzuki N (2022). Nationwide cross-sectional survey of patients with relapsing polychondritis in 2019 demonstrates reduction of airway involvement compared with that in 2009. Sci. Rep..

[16] Sangle SR (2023). Relapsing polychondritis: A single centre study in the United Kingdom. Autoimmun. Rev..

